# A Cambrian unarmoured lobopodian, †*Lenisambulatrix humboldti* gen. et sp. nov., compared with new material of †*Diania cactiformis*

**DOI:** 10.1038/s41598-018-31499-y

**Published:** 2018-09-20

**Authors:** Qiang Ou, Georg Mayer

**Affiliations:** 1 Early Life Evolution Laboratory, State Key Laboratory of Biogeology and Environmental Geology, China University of Geosciences (Beijing), Beijing, 100083 China; 2 Department of Zoology, University of Kassel, 34132 Kassel, Germany

## Abstract

Cambrian marine lobopodians are generally considered as predecessors of modern panarthropods (onychophorans, tardigrades, and arthropods). Hence, further study of their morphological diversity and early radiation may enhance our understanding of the ground pattern and evolutionary history of panarthropods. Here, we report a rare lobopodian species, †*Lenisambulatrix humboldti* gen. et sp. nov. (“Humboldt lobopodian”), from the early Cambrian Chengjiang Lagerstätte and describe new morphological features of †*Diania cactiformis*, a coeval armoured lobopodian nicknamed “walking cactus”. Both lobopodian species were similar in possessing rather thick, elongate lobopods without terminal claws. However, in contrast to †*Diania cactiformis*, the body of which was heavily armored with spines, the trunk and limbs of the Humboldt lobopodian were entirely unarmored. Our study augments the morphological diversity of Cambrian lobopodians and presents two evolutionary extremes of cuticular ornamentation: one represented by the Humboldt lobopodian, which was most likely entirely “naked”, the other epitomized by †*D. cactiformis*, which was highly “armoured”.

## Introduction

Lobopodians were marine, caterpillar-like Palaeozoic animals characterized by non-segmented limbs called lobopods or lobopodia (singular: lobopodium; from Greek *λοβός* [*lobos*], rounded projection or protuberance; and *ποδός* [*podos*], foot). Lobopodians originated and rapidly diversified^1^ during the Cambrian radiation of metazoan body plans and their marine representatives survived at least until the end of the Carboniferous Period^2^. Lobopodians are generally considered as a paraphyletic assemblage because some were most likely the forerunners of modern lobopod-bearing animals^3–5^, the onychophorans and tardigrades, and their closest relatives, the arthropods. Hence, lobopodians are pivotal for understanding the origin and early evolution of the total-group Panarthropoda.

Lobopodians are taxonomically diverse, with over 30 species described to date (Table 1). Their rapid radiation and adaptation to various ecological niches were documented in particular in Cambrian Lagerstätten (Stage 2 to Drumian; Table 1). Recent phylogenetic analyses resolved the clustering of (i) hallucigeniids (including representatives of †*Hallucigenia*, †*Cardiodictyon*, †*Carbotubulus*, and probably also †*Microdictyon*) characterized by one to three pairs of modified (slender, tentacle-like) anterior appendages and a well-differentiated head^5^; (ii) luolishaniids (including representatives of †*Luolishania*, †*Collinsium*, †*Acinocricus*, “Collins’ monster”, and probably also †*Facivermis*) distinguished by pairs of modified (elongated, spinous, suspension-feeding) anteriormost and even specialized posteriormost appendages (e.g., †*Ovatiovermis cribratus*)^3,5^; and (iii) the “large lobopodians” (representatives of †*Jianshanopodia*, †*Megadictyon*, †*Hadranax*, †*Kerygmachela*, †*Pambdelurion*, and †*Siberion*), which comprise a paraphyletic assemblage closely related to arthropods^3,5^. These three groups show varying degrees of appendage specialization and tagmosis (body tagmatization).

**Table 1:** List of described species of marine lobopodians with soft-bodied preservation.

No.	Species*	References	Lagerstätte	Horizon
1	†*Xenusion auerswaldae*	Pompeckj^32^; Dzik & Krumbiegel^33^	Kalmarsund sandstone	Cambrian, Stages 2–3
2	†*Antennacanthopodia gracilis*	Ou *et al*.^21^	Chengjiang mudstone	Cambrian, Stage 3
3	†*Cardiodictyon catenulum*	Hou *et al*.^34^		
4	†*Diania cactiformis*	Liu *et al*.^25^; Ma *et al*.^26^		
5	†*Hallucigenia fortis*	Hou & Bergström^35^		
6	†*Jianshanopodia decora*	Liu *et al*.^36^		
**7**	**†*Lenisambulatrix humboldti*** **gen. et sp. nov**.	**Present study**		
8	†*Luolishania longicruris*	Hou & Chen^37^; Ma *et al*.^20^		
9	†*Facivermis yunnanicus*	Hou & Chen^38^; Liu *et al*.^39^		
10	†*Megadictyon haikouensis*	Luo *et al*.^40^; Liu* et al*.^41^		
11	†*Microdictyon sinicum*	Chen *et al*.^42^; Chen *et al*.^28^		
12	†*Onychodictyon ferox*	Ramsköld & Hou^43^; Ou *et al*.^17^		
13	†*Onychodictyon gracilis*	Liu *et al*.^44^		
14	†*Paucipodia inermis*	Chen *et al*.^45^; Hou *et al*.^6^		
15	†*Collinsium ciliosum*	Yang *et al*.^3^	Xiaoshiba mudstone	
16	†*Tritonychus phanerosarkus*	Zhang *et al*.^46^	Orsten-type (China)	
17	†*Hadranax augustus*	Budd & Peel^47^	Sirius Passet shale	
18	†*Kerygmachela kierkegaardi*	Budd^48^; Budd^49^		
19	†*Pambdelurion whittingtoni*	Budd^50^; Vinther *et al*.^51^		
20	†*Siberion lenaicus*	Dzik^52^	Sinsk “algal lens”	Cambrian, Stage 4
21	†*Hallucigenia hongmeia*	Steiner *et al*.^53^	Guanshan mudstone	
22	†*Collinsium* sp.	Jiao *et al*.^54^		
23	“Collins’ monster”	García-Bellido *et al*.^55^	Emu Bay Shale	
24	†*Acinocricus stichus*	Conway Morris^56^	Spence Shale	Cambrian, Wuliuan
25	“Collins’ monster”	Collins^57^	Burgess Shale	
26	†*Aysheaia pedunculata*	Whittington^16^		
27	†*Hallucigenia sparsa*	Smith and Caron^19^		
28	†*Ovatiovermis cribratus*	Caron and Aria^5^		
29	†*Orstenotubulus evamuellerae*	Waloszek^58^; Maas *et al*.^59^	Orsten-type (Sweden)	Cambrian, Drumian
30	Unnamed luolishaniid	Van Roy *et al*.^60^	Fezouata mudstone	Ordovician, Tremadocian
31	Unnamed xenusiid	Whittle *et al*.^61^	Soom Shale	Ordovician, Hirnantian
32	†*Carbotubulus waloszeki*	Haug *et al*.^2^	Mazon Creek concretions	Upper Pennsylvanian

^*^Records of ambiguous or controversial species^29,39,62–64^ are not included. We endorse the lobopodian affinity of †*Facivermis yunnanicus*^5^ and retain †*Onychodictyon gracilis*, although the validity of this species has been challenged^10^.

Here, we describe a new taxon, †*Lenisambulatrix humboldti* gen. et sp. nov., which arguably shows the lowest degree of modification of appendages and the absence of ornamentation on trunk and appendages, thus resembling †*Paucipodia inermis* from the same Lagerstätte^6^. Moreover, we provide new data on the cephalic morphology and report the occurrence of modified appendages in the heavily armoured lobopodian species †*Diania cactiformis*.

## Results

### Systematic paleontology

Total group Panarthropoda Nielsen (1995)^7^

“Lobopodia” Boudreaux (1979)^8^

†Lenisambulatrix gen. nov.

#### Type species

†Lenisambulatrix humboldti gen. et sp. nov., by monotypy.

#### Genus etymology

Latin *lenis*, soft, smooth, or gentle, alluding to the unarmored body; *ambulatrix*, walker^9^. Gender: feminine.

#### Genus diagnosis

Lobopodian panarthropod characterized by an entirely unornamented body. Tubular trunk metamerically segmented, with at least eight homonymous segments. Each segment bearing a pair of long, thick, lobopodal limbs devoid of ornaments and terminal claws. Trunk gradually widening and gently tapering towards one end, probably forming a head region.

**Figure 1:**
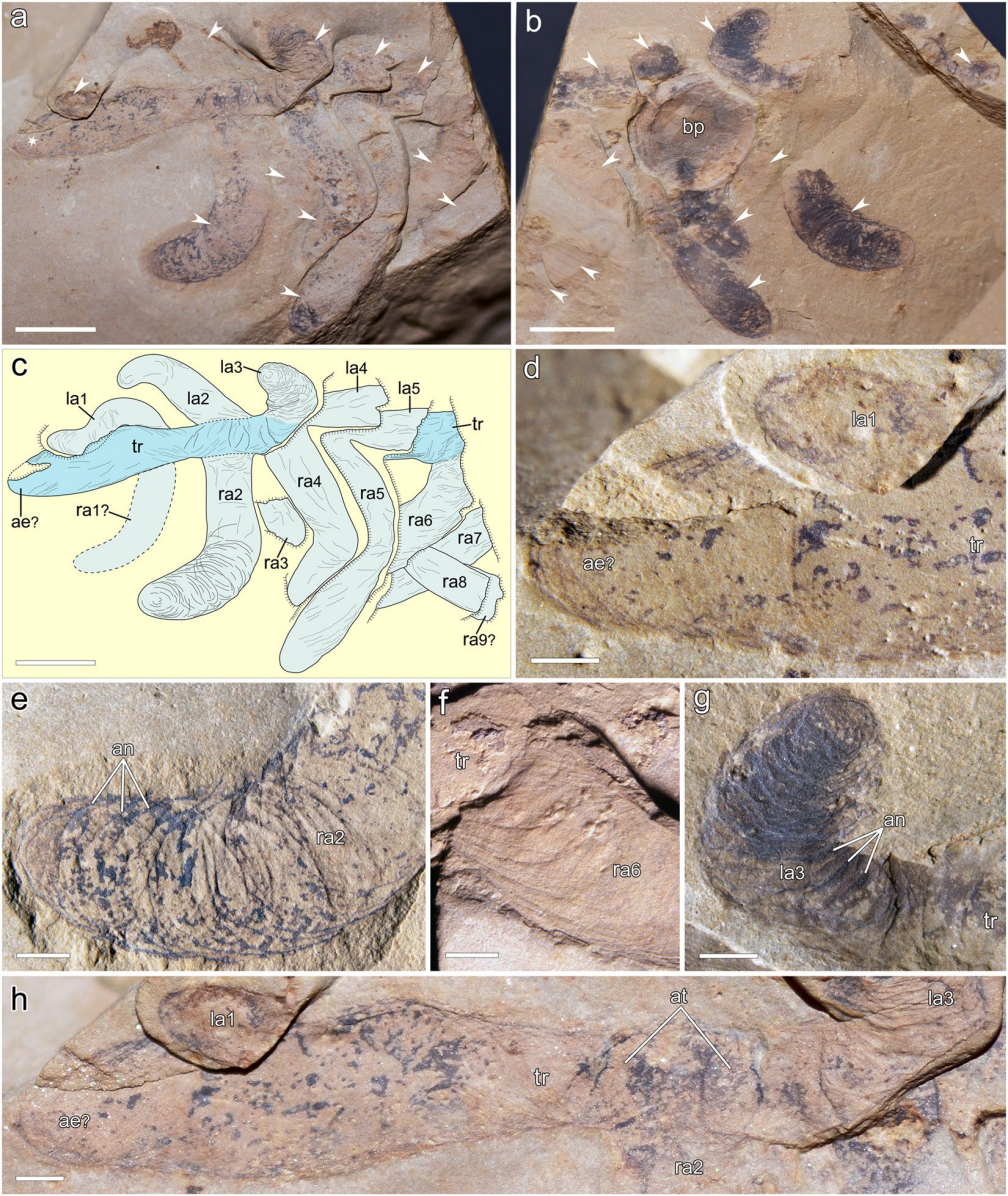
Holotype specimen (ELEL-SJ080744) of †*Lenisambulatrix humboldti* gen. et sp. nov. (**a**) Part showing paired thick lobopods (arrowheads) attached to the trunk. Asterisk indicates a body terminus interpreted as the anterior end. (**b**) Counterpart showing lobopods (arrowheads) largely preserved as carbonaceous films. Annuli are evident on bent appendages. (**c**) Composite interpretive drawing of part and counterpart. Appendages in pale grey; trunk in light blue; sediment escarps indicated by comb-like lines; uncertain boundaries by dashed lines. (**d**) Close-up of the almost featureless anterior end and an incomplete appendage. (**e**) Distal part of the second right lobopod showing a bent posture and composite imprint of annuli. (**f**) Proximal part of the sixth right lobopod attached to the trunk. (**g**) Close-up of the flexed third left lobopod with evident annuli. (**h**) Close-up of trunk and its presumed anterior extension. Abbreviations: ae, presumed anterior end; an, annuli; at, attachment sites of lobopods; bp, associated brachiopod shell; la1–la5, left lobopods 1 to 5; ra1–ra9, right lobopods 1 to 9; tr, trunk. Scale bars: 5 mm (**a**–**c**); 1 mm (**d**–**h**).

**Figure 2:**
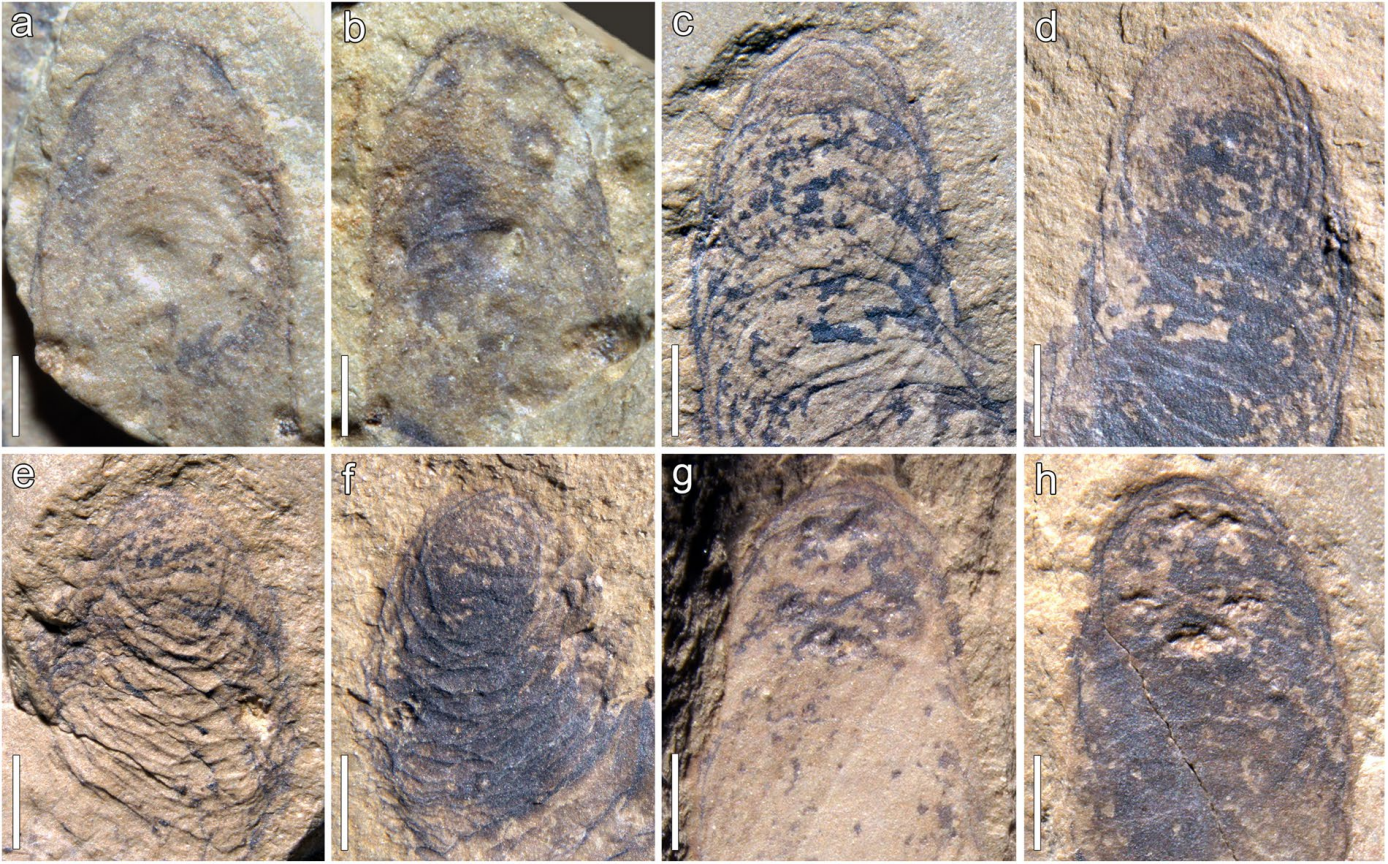
Distal portion of lobopods of †*Lenisambulatrix humboldti* gen. et sp. nov. (**a**,**b**) Part and counterpart of the first left lobopod (la1). (**c**,**d**) Part and counterpart of the second right lobopod (ra2). (**e**,**f**) Part and counterpart of the third left lobopod (la3); note the compressed and deformed annuli. (**g**,**h**) Part and counterpart of the fifth right lobopod (ra5); note the irregular, raised nodes occurring on the distal end (and also on other portions of other lobopods) as taphonomic artifacts. Scale bars: 500 µm (**a**,**b**); 1 mm (**c**–**h**).

†*Lenisambulatrix humboldti* gen. et sp. nov. (Figs 1 and 2).

#### Species etymology

In honour of Friedrich Wilhelm Heinrich Alexander von Humboldt, in recognition of his contribution to the natural sciences, as well as in honour of the Alexander von Humboldt Foundation (AvH), which supported the present work.

#### Species diagnosis

As for the genus.

LSID urn:lsid:zoobank.org:pub:3D495AE4-3D1C-4750-8730-57BD6B38BCAF

LSID urn:lsid:zoobank.org:act:F3FF6A37-4368-40E5-965F-D004BEC23781

#### Material

Single specimen, ELEL-SJ080744 (with part and counterpart).

#### Occurrence and stratigraphy

Lower part of the †*Eoredlichia*–†*Wutingaspis* Biozone, upper Yu’anshan Member, Heilinpu Formation, Cambrian Series 2, Stage 3 (~520 Ma). The new species occurred in the horizon that also yielded †*Diania cactiformis* in the Huaguoshan section, Erjie^10^, Kunming, Yunnan, South China.

#### Remarks

Numerous authors cite Snodgrass (1938)^11^, although in fact he did not introduce the taxon “Lobopodia”. See also discussion by Ortega-Hernández (2016)^12^. According to our inquiries, the earliest author using this taxonomic name with reference to Snodgrass (1938)^11^ was Boudreaux (1979)^8^. We have included “Lobopodia” in quotation marks because it refers to a non-monophyletic assemblage^4^. †*Lenisambulatrix humboldti* gen. et sp. nov. differs from other lobopodians in having an entirely unadorned body (without setae or sclerotized elements such as spines and plates) and exceptionally thick, smooth lobopods without terminal claws.

#### Preservation and taphonomy

The specimen was preserved primarily as dark, carbonaceous and aluminosilicate films in a weathered yellowish argillaceous mudstone. In places, original tissues and cuticle were replaced by dark red to brown iron (III) oxide fine grains that resulted from weathering of microscopic diagenetic pyrite. Pyrite framboids and euhedral crystals precipitated and disseminated particularly along the outline or on the surface of the lobopods and the trunk during later diagenetic processes^13^. Cuticular annuli or folds of trunk appendages were preserved as composite imprints of sub-circular fine grooves and ridges. The specimen was oblique-ventrally compressed, with anterior pairs of trunk appendages splaying upward and crosscutting sediment laminae. Posterior pairs of lobopods are preserved in successive sediment layers, with each pair approximately parallel to the bedding surface. Such exceptional preservation of soft-bodied tissues suggests this animal was rapidly buried by fine sediment in a catastrophic event^14^. A specimen of the brachiopod †*Diandongia pista*^15^ was coincidentally buried together with the specimen of †*Lenisambulatrix humboldti* gen. et sp. nov. and superimposed on the mid-ventral portion of the latter.

### Description of †*Lenisambulatrix humboldti* gen. et sp. nov

#### General morphology

This animal shows a simple anatomy and is soft-bodied, without any evidence of hard parts such as sclerotized plates or spines. It bears a vermiform trunk that consists of at least eight metameric segments, each with a pair of thick, long lobopods (Fig. 1a–c). The animal was incompletely preserved, with the preserved body part ~29.4 mm in length.

#### Body termini

Only one end of the body was preserved. It protrudes beyond the first lobopod pair as an elongate, rod-like structure. It extends from the trunk stem, gradually widens, and gently tapers towards the distal end. Other than some obscure wrinkles occurring along its margin, this body end is almost featureless and shows no structures that could be interpreted as appendages (Fig. 1d). Provisionally, we designate it the anterior end (see discussion below).

#### Trunk

There is no evidence of tagmosis in the elongate trunk, which consists of a longitudinal series of at least nine homonomous segments (Fig. 1a–c). The anterior portion of the trunk is better preserved than the posterior one, which is bent downward and was buried in lower sediment laminae (Fig. 1c). The width of the preserved trunk varies from 2.0 to 3.4 mm. There are no evident annuli preserved on the trunk surface, but some wrinkles appear around the attachment sites of limbs (Fig. 1a,c). There is no evidence of internal anatomical structures, such as a gut, muscles, or body cavity, in the trunk.

#### Appendages

There are at least nine pairs of long, thick, unjointed appendages (lobopods) associated with the trunk. The lobopods are largely preserved as dark imprints of carbonaceous films (original kerogen), although remains of one lobopod (la2, revealed after excavation) are represented by iron oxides along its margin and at the distal portion (Fig. 1a). The three anterior pairs of lobopods are splayed on both sides and bent upward to varying degrees (Fig. 1a–c), with one lobopod (la1) partially overlying the anterior trunk, which indicates a ventral view. At the distal portion of lobopods that are bent both upward and anteriorly (e.g., ra2 and la3; Fig. 1e,g), closely spaced annuli occur as composite imprints of curved, shallow furrows and narrow ridges (Fig. 2c–f). Besides, sparse annuli appear at the proximal portion of the lobopods (e.g., Fig. 1f). Mechanical removal of the shell of the brachiopod †*Diandongia pista* from the part revealed the paired arrangement of lobopods (including the 4^th^ and 5^th^ pairs; Fig. 1a,c). Measured from the best preserved lobopod (ra5), which was compressed parallel to the bedding plane, the length reaches 18.0 mm. The maximum width occurs at the middle portion of lobopods, measuring ~3.6 mm in thickness; the width decreases distally, forming a rounded end. There is no evidence of papillae, spines, or terminal claws associated with the lobopods (Fig. 2a–h).

### Description of new material of †*Diania cactiformis* Liu *et al*., 2011

#### General morphology

**Figure 3:**
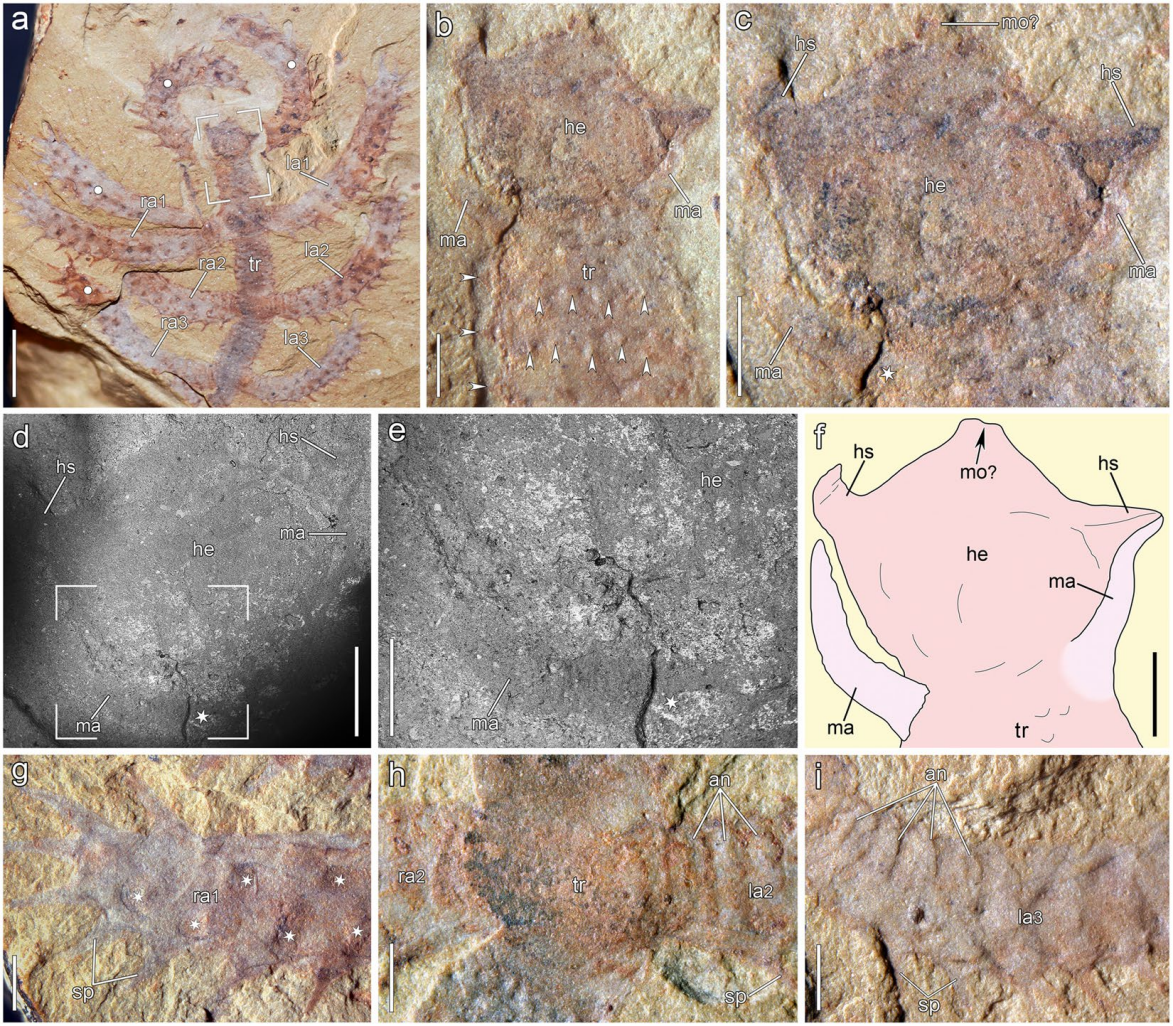
Details of †*Diania cactiformis* based on new material. (**a**) Incomplete, ventrally compacted specimen (ELEL-SJ102058) showing anterior trunk segments terminated by a possible head. Lobopods of another overlying individual are marked by white circles. (**b**) Close-up of focus area in (**a**) showing a putative head with lateral spines and modified appendages. Arrowheads indicate trunk spines. (**c**) Details of anterior structures, including the helmet-like head and a pair of modified appendages. Asterisk (in c, d, e) indicates attachment site of a modified appendage. (**d**,**e**) Backscattered electron (BSE) analysis of anterior structures (**d**) and details (**e**) of a modified appendage (focus area in d). Dark areas in BSE images suggest soft tissues preserved as organic carbon residues; bright areas suggest pyrites. (**f**) Interpretative drawing of (**c**). (**g**) Distal part of the first right lobopod. Asterisks indicate imprints of spine bases. (**h**) Second trunk segment showing proximal part of lobopods with annuli. (**i**) Proximal part of the third left lobopod showing annuli. Abbreviations: an, annuli; he, head; hs, head spine; ma, modified appendage; la1–la3, left lobopods 1 to 3; ra1–ra3, right lobopods 1 to 3; sp, spine; mo, mouth; tr, trunk. Scale bars: 5 mm (**a**); 1 mm (**b**–**d**,**f**–**i**); 500 µm (**e**).

The new specimen of †*Diania cactiformis* (ELEL-SJ102058; Fig. 3a–i), although incomplete, shows some novel morphological details. The body surface of this lobopodian is almost completely covered with rigid spines, including the head, trunk and lobopods. The specimen was ventrally compressed. The anterior end is differentiated into a distinctive helmet-like structure, which is succeeded by a slender portion of the trunk (Fig. 3a). The three preserved trunk segments are homonomous, each bearing a pair of long, thick, spinous lobopods. The preserved body part measures ~22.1 mm in length.

#### Head and modified anterior appendages

Anterior to the first pair of walking appendages, the trunk continues into a terminal, helmet-like structure interpreted here as the head (Fig. 3a–f). The head is slanted sideward (Fig. 3a,b), indicating its movability. The helmet-like structure of the head measures 4.4 mm in maximum width and 3.2 mm in length and shows a semicircular posterior margin delineating it from the trunk. The median part of the head is characterized by a pair of laterally tapered structures, interpreted here as lateral head spines. The distal part of the head shows a cone-shaped terminal structure, most likely representing the buccal region with a terminal mouth (Fig. 3b,c,f). A pair of unjointed, flexible, tentacle-like structures (~2.7 mm in length), which were revealed after preparation, project from the base of the head (Fig. 3b–f). These structures are interpreted here as a pair of modified sensory appendages. Although these appendages topologically belong to the trunk, they are thinner and shorter than the remaining trunk lobopods. Moreover, in contrast to the walking lobopods of the trunk they are not armored with spines. The exposed attachment site of the right appendage (asterisk in Fig. 3c–e), suggests that the specimen is exposed in ventral view.

#### Trunk

The preserved trunk in the specimen is slender and column-like, showing no evidence of tagmosis (Fig. 3a). The trunk gently tapers posteriorly, with its width ranging from 3.0 mm (1^st^ segment) to 2.6 mm (3^rd^ segment). Numerous short, spinous projections occur on the lateral and ventral (and most likely also on the dorsal) sides of each interpedal trunk region, arrayed in evenly spaced transverse rows. The number of spine rows per interpedal region increases from three between the modified appendages and the first pair of armored lobopods (Fig. 3b) to at least five further posteriorly. Succeeding rows of spines are probably arranged in a staggered pattern (Fig. 3b). The trunk portions where the lobopods insert show no evidence of spines (Fig. 3h). Ornaments of trunk surface, such as cuticular annuli, nodes, or papillae, are lacking. Internal anatomies of the trunk, such as the alimentary canal, muscles, or body cavity, are not seen in the specimen.

#### Walking lobopods

The walking lobopods are long, thick, rod-like structures equipped with numerous sclerotized spines. The length of the lobopods decreases posteriorly from 17.9 mm (1^st^ pair) and 14.7 mm (2^nd^ pair), to 11.6 mm (3^rd^ pair). The proximal portion of lobopods (attached to the trunk) shows the minimum width, ranging from 2.3 mm (1^st^ pair), 1.9 mm (2^nd^ pair), to 1.8 mm (3^rd^ pair). The maximum width is measured in the middle portion of each lobopod, varying from 2.7 mm (1^st^ pair) to 2.2 mm (2^nd^ pair). The robust, long spines or spinous cuticular projections are distributed throughout the lobopod surface except for the proximal region (Fig. 3h,i). They project from a broad base and taper distally to a pointed end, with a maximum length of ~1.6 mm at the distal end of the lobopod (Fig. 3g). The spines are probably arranged in longitudinal rows (Fig. 3a,g), but the number of rows cannot be determined in the specimen. Integumental annuli (~5 in number) are evident only in the proximal region of each lobopod. The specimen provides no indication of papillae or terminal claws associated with the lobopods.

## Discussion

### Anteroposterior orientation

**Figure 4:**
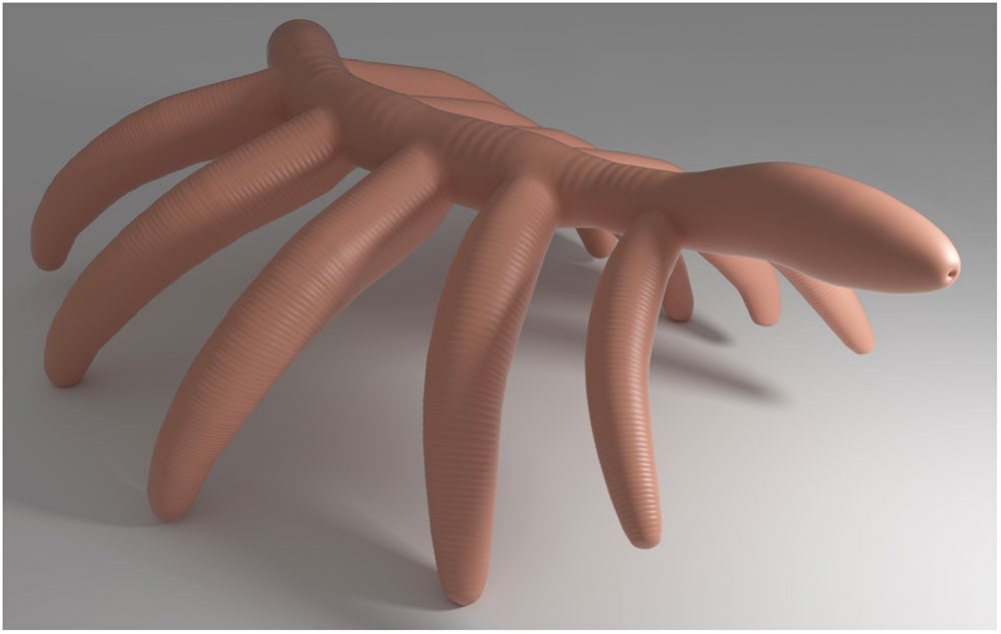
Three-dimensional reconstruction of †*Lenisambulatrix humboldti* gen. et sp. nov. in life position. Anteroposterior orientation, terminal mouth, and surface color are conjectural.

The anteroposterior orientation of †*Lenisambulatrix humboldti* gen. et sp. nov. (Fig. 4) remains conjectural, with only one body terminus preserved. This terminus lacks any distinctive features, such as proboscis, mouth, eyes, tentacle-like appendages or an unpaired sclerite, which would be indicative of a head. Nor is there any unambiguous indication from the trunk, such as tagmosis, modified anterior appendages, or claw direction, which would hint at the anteroposterior orientation of the specimen. Nonetheless, we tentatively interpret the preserved body terminus as the anterior end. This is informed by two aspects. First, the considerable expansion some distance away from the distal end of the body terminus is reminiscent of Cambrian lobopodians with an expanded head region, such as †*Cardiodictyon catenulum* and †*Hallucigenia fortis*. Second, most other Cambrian lobopodians possessed an elongate anterior end and lacked a posterior trunk extension, including at least †*Aysheaia pedunculata*^16^, †*Onychodictyon ferox*^17,18^, †*Hallucigenia sparsa*^19^, †*Ovatiovermis cribratus*^5^, and †*Microdictyon sinicum*^1,10^, whereas the alleged short posterior extension of †*Collinsium ciliosum*^3^ and †*Luolishania longicruris*^20^ has been questioned^5^. The only exceptions occur in †*Paucipodia inermis*^6^ and †*Antennacanthopodia gracilis*^21^, which seem to have a comparatively long posterior extension behind the last pair of lobopods. However, the posterior extension of these two species lacked a significant expansion which occurred on the preserved body terminus of †*Lenisambulatrix humboldti* gen. et sp. nov. Among extant descendants of Palaeozoic lobopodians, tardigrades lack a posterior trunk extension^22^, whereas onychophorans possess an anal cone, which is a true, limbless segment^23,24^.

Complete specimens of the lobopodian †*Diania cactiformis* show comparatively long trunk extensions at both body termini, thus leaving open the anteroposterior orientation of this fossil^25,26^. Our findings of a distinctive, helmet-like structure with a pair of lateral spines, and a putative terminal mouth at one terminus, as well as a pair of modified appendages at the junction between the helmet-like structure and the trunk, suggest that this was the anterior end of the animal. The mouth of †*Diania cactiformis* was most likely terminal and pointed anteriorly in life, like the mouth of *Onychodictyon ferox*^17^ and *Aysheaia pedunculata*^16^. However, we cannot exclude the possibility that it was directed more or less ventrally in life (as in *Hallucigenia sparsa*^19^), but it might have turned upward during burial. The terminal, bulbous structure seen in Figs 1D and 3D,E of Ma *et al*.^26^ most likely corresponds to the helmet-like head structure in our material.

### Comparison

The most striking similarity between †*Lenisambulatrix humboldti* gen. et sp. nov. and †*Diania cactiformis* are their extraordinarily thick trunk appendages (lobopods). These are obviously thicker than the equivalents of other Palaeozoic lobopodians. Unlike most other lobopodians, including representatives of †*Onychodictyon*, †*Hallucigenia*, †*Microdictyon*, †*Paucipodia*, †*Tritonychus*, and †*Collinsium*, that bore terminal claws, such structures are absent in both species studied herein. The cuticular spines on the distal portion of each lobopod of †*Diania cactiformis* are reminiscent of terminal claws. However, these structures are fanned out and are indifferent from the rest spines on other portions of the lobopods in morphology and preservation. On the other hand, they are clearly different from the terminal claws of other lobopodians (e.g., †*Onychodictyon ferox*^17^ and †*Paucipodia inermis*^27^) which showed more rigidity and were generally curved, rooted in the lobopod and linked via an internal tendon. In addition to the lack of true claws and given that the preserved body terminus of †*Lenisambulatrix humboldti* gen. et sp. nov. is indeed the anterior end, both species have two other features in common: (i) they share an elongate, expanded anterior end with a similar ratio of anterior end length to maximum appendage length (0.45 vs. 0.40); and (ii) the maximum trunk width occurs roughly in the middle of the anterior end of both species.

Nevertheless, these two species do differ in several respects. First, the head of †*Diania cactiformis* is clearly delineated from the trunk; it bears a pair of large lateral spines and is immediately succeeded by a pair of modified appendages. In contrast, the anterior end of †*Lenisambulatrix humboldti* gen. et sp. nov. appears almost featureless. Second, †*Diania cactiformis* is heavily armored with sclerotized spines covering the trunk, the lobopods and even the head, whereas at least the preserved part of †*Lenisambulatrix humboldti* gen. et sp. nov. is completely soft bodied. Third, the lobopods of †*Diania cactiformis* show clear annulation only in the proximal region, whereas annuli are particularly evident in the distal part of lobopods in the Humboldt lobopodian. Fourth, the maximum appendage width of †*Lenisambulatrix humboldti* gen. et sp. nov. is greater than its maximum trunk thickness (provided that it was not increased by taphonomic factors), whereas the reverse is the case in †*Diania cactiformis*.

†*Lenisambulatrix humboldti* gen. et sp. nov. and †*Diania cactiformis* apparently share an elongated anterior end with some other Cambrian lobopodians, including †*Microdictyon sinicum*^28^, †*Paucipodia inermis*^6^, and †*Hallucigenia sparsa*^19^. The robust lateral head spines of †*Diania cactiformis* are reminiscent of the paired head sclerites in †*Collinsium ciliosum*^3^ and the lateral spinous head sclerites in †*Luolishania longicruris*^29^.

### Anterior appendage specialization

The anteriormost pair of appendages of †*Diania cactiformis*, modified as short tentacles and situated directly behind the helmet-like head, are much shorter and thinner than the remaining trunk lobopods. This condition is comparable to that in representatives of †*Hallucigenia*, †*Cardiodictyon*, and probably also †*Carbotubulus*, which have been grouped together in Hallucigeniidae *sensu lato*^5^ and are characterized by one to three anterior pairs of slenderized, tentacle-like appendages. Likewise, representatives of Luolishaniidae, including †*Luolishania longicruris*, “Collins’ monster”, †*Collinsium ciliosum*, and †*Ovatiovermis cribratus*, show a distinct specialization of anterior appendages, which are otherwise elongated and setaceous, probably adapted for suspension feeding^5^. Hence, †*Diania cactiformis* might be closely related to hallucigeniids and luolishaniids. In contrast, †*Lenisambulatrix humboldti* gen. et sp. nov., like †*Paucipodia inermis*, shows no appendage specialization and a low degree of body tagmosis in the anterior body section (Fig. 4), which would suggest a basal position in the panarthropod tree.

### Sclerotization and arthropodization

†*Lenisambulatrix humboldti* gen. et sp. nov. shows no sign of sclerotized structures and also no evidence of arthropodization of limbs or arthrodization of the trunk. In the new specimen of †*Diania cactiformis*, the maximum thickness of the trunk is slightly greater than that of the appendages. However, Ma *et al*.^26^ reported that the appendages of †*Diania cactiformis* were thicker than the trunk. Rather than a taphonomic artifact, this discrepancy might have resulted from the localized contraction/expansion of the trunk/limbs and suggests that †*Diania cactiformis* was fundamentally a soft-bodied animal without an exoskeleton. In contrast, the sclerotized armature (exoskeleton) of most arthropods does not allow contraction or expansion that would change the thickness of trunk or limbs, rejecting a taphonomic origin of this condition. Soft deformations (i.e., curvature and annulation of the limbs and trunk) also indicate the possession of a hydrostatic skeleton^27^ and lack of an exoskeleton in †*Diania cactiformis*. Nonetheless, the rigid, robust spines of this species indicate that its integument was thickened and hardened in places, although it may be inappropriate to term these spines sclerites. In contrast to previous descriptions^25,30^, our new material of †*Diania cactiformis* shows no evidence of sclerotized segments in the trunk lobopods. Nor are there any articulating structures (joints), such as pivots, condyles, or less-sclerotized membranous hinges in our specimen. This is particularly obvious in the anteriormost pair of modified lobopods, which lack spines. Thus, our observations support the conclusion that unequivocal arthropodization was absent in †*Diania cactiformis*^26^. This means that †*Diania cactiformis* cannot provide valuable insights into the early evolution of arthropod limbs.

### Locomotion and life mode

The thick lobopods of †*Lenisambulatrix humboldti* gen. et sp. nov. and †*Diania cactiformis* do not show any terminal claws and might have been adapted for walking or crawling on soft substrates of the Cambrian seafloor. The conspicuous armature of †*Diania cactiformis* most likely served for protection from predators. In contrast, the presumed entirely soft-bodied lobopodian †*Lenisambulatrix humboldti* gen. et sp. nov. may have led a reclusive life, probably hiding among substrate crevices or clusters of sponges to avoid predators. Given the lack of modified anterior appendages and mouthparts, this species might have been a deposit feeder or a scavenger. The modified appendages of †*Diania cactiformis* might have functioned as sensory antennae or facilitated feeding, coupled with the remarkable flexibility of its head. The close association of two individuals of †*Diania cactiformis* in our material, with one directly overlain by the other, is either a coincidence or evidence for a rapid burial during copulation.

## Conclusion

The presumably soft-bodied new species, †*Lenisambulatrix humboldti* gen. et sp. nov., extends our knowledge of the taxonomic composition and morphological diversity of lobopodians. This species is comparable to the coeval and sympatric species †*Diania cactiformis* in that both shared extraordinarily thick, long, homonomous walking lobopods. Furthermore, our study revealed a pair of modified anterior appendages in †*Diania cactiformis*, which allies this lobopodian closely to hallucigeniids and luolishaniids. Our findings further corroborate the previous assumption^26^ that arthropodization was lacking in †*Diania cactiformis*. This highly armoured species continues to be phylogenetically important albeit controversial^3,5,25,26,31^. Our study of the rare material of †*Lenisambulatrix humboldti* gen. et sp. nov. reveals a relatively simple organization of this lobopodian, which might indicate a basal position in the panarthropod tree.

## Materials and Methods

Only a single specimen of †*Lenisambulatrix humboldti* gen. et sp. nov. was recovered from the Chengjiang deposits during the past nine years (2008–2017). It was yielded from the Huaguoshan section, Sanjiezi village, Erjie town^10^ (~50 km west of the classic Maotianshan section in the vicinity of Chengjiang County), Kunming, Yunnan. One new specimen of †*Diania cactiformis* (ELEL-SJ102058) was collected in 2010 from the same locality and horizon. Both specimens were deposited in the Early Life Evolution Laboratory (ELEL), China University of Geosciences, Beijing. Mechanical preparations were performed using a Stemi 508 stereomicroscope (Carl Zeiss MicroImaging GmbH, Jena, Germany) under various light conditions. The specimen was photographed using a Nikon D7000 camera under sunlight and an Axio Zoom V16 stereomicroscope equipped with an Axiocam 503 color digital camera (Carl Zeiss MicroImaging GmbH). Backscattered electron (BSE) analysis was performed in Key Laboratory of Orogenic Belts and Crustal Evolution, Peking University, China, using an FEI Quanta 650 FEG scanning electron microscope (SEM) in low-vacuum mode (50 Pa) with accelerating voltage of 10 keV and emission current of 290 µA. Morphological measurements were conducted using the Carl Zeiss AxioVision 4.9.1.0 software package. Three-dimensional reconstruction of the new species was conducted using the free and open 3D creation software Blender 2.78.
